# Introduction to Editorial Board Member: Professor Paula T. Hammond

**DOI:** 10.1002/btm2.10085

**Published:** 2018-01-19

**Authors:** Nisarg J. Shah

**Affiliations:** ^1^ John A. Paulson School of Engineering and Applied Sciences, Harvard University Cambridge MA 02138

In this issue of *Bioengineering and Translational Medicine*, we are pleased to introduce our Editorial Board Member, Professor Paula T. Hammond. Prof. Hammond is the David H. Koch Chair Professor of Engineering and the Head of the Department of Chemical Engineering at the Massachusetts Institute of Technology (MIT). Prof. Hammond received her BS degree from MIT in Chemical Engineering, her MS degree from the Georgia Institute of Technology, and her PhD degree from MIT.

Over the past two decades, Professor Hammond's research has made deep and impactful contributions in the fields of synthetic polymers, nanomedicine, and materials assembly. Professor Hammond is a leader in the use of molecular interactions, which include electrostatic and hydrogen‐bonding forces, to generate “layer‐by‐layer” deposited functional materials with highly controlled microscale and nanoscale structures on a broad range of surfaces as a means to create ordered architecture.^1^, ^2^, ^3^ Her research has leveraged these nanoscale forces, inherent to many synthetic and natural materials, toward developing a range of new biochemical and electrochemical materials with synergistic functionality that are well beyond the capacity of individual materials.

Professor Hammond's research draws on and extends concepts of thermodynamics and self‐assembly in which the basis of the approach is the use of secondary, or nonspecific interactions, in combination with steric repulsion and electrostatic interactions, to direct the deposition of molecules and larger scale materials systems onto activated surfaces in a stepwise layer‐by‐layer approach. She has pioneered the development and application of this technology to generate controlled release thin film coatings for biomedical implants that address tissue regeneration,^4^ wound healing,^5^ and transdermal delivery from microneedle platforms,^6^ to nanoparticle drug carriers for drug, gene, and siRNA delivery.^7^, ^8^, ^9^ Professor Hammond has also extended the applications of self‐assembled materials to electrochemical energy devices,^10^ including photovoltaics^11^ and fuel cells.^12^

Another major research area in Professor Hammond's group focuses on nanoscale self‐assembly through the design of functionalized block copolymers. Block copolymers, which consist of two or more covalently bound polymer segments of different chemical composition, are known for their ability to microphase separate and organize into mesophase structures on nanometer length scales in the bulk state, and at surfaces and interfaces, based on chemical differences between blocks. Professor Hammond's research has demonstrated the role of controlling the molecular architecture on the nanoscale in the ordering of block copolymer morphology, particularly for copolymer systems with asymmetric (irregular or nonlinear) blocks. Professor Hammond's research has investigated the development and application of dendritic‐linear block copolymers as nanoencapsulants for targeted drug delivery.^13^

Professor Hammond has led a remarkable career as a researcher and educator. Her research has been published in over 300 journal articles and book chapters, and has been heavily cited, with more than 21,000 citations. In recognition of these contributions to materials chemistry and nanomedicine, Prof. Hammond has been presented with numerous awards and honors. She has been elected to the National Academy of Engineering (2017), the National Academy of Medicine (2016), and the American Academy of Arts and Sciences (2013). She is an elected Fellow of the American Physical Society, the American Institute of Biological and Medical Engineers, and the American Chemical Society Polymer Division. She is the recipient of the AIChE Charles M. A. Stine Award (2013) in recognition of outstanding contributions to the field of materials science and engineering, and the Alpha Chi Sigma Award (2014) for Chemical Engineering Research. Professor Hammond has been featured numerous times in the top 100 cited materials scientists. In addition to research and teaching excellence, Prof. Hammond has had a deep interest for administrative leadership. At MIT, she co‐founded the MIT Institute for Soldier Nanotechnology. She served as the Executive Officer of the Chemical Engineering Department of MIT (2008–2011) and currently serves as the department chair.

**Figure 1:**
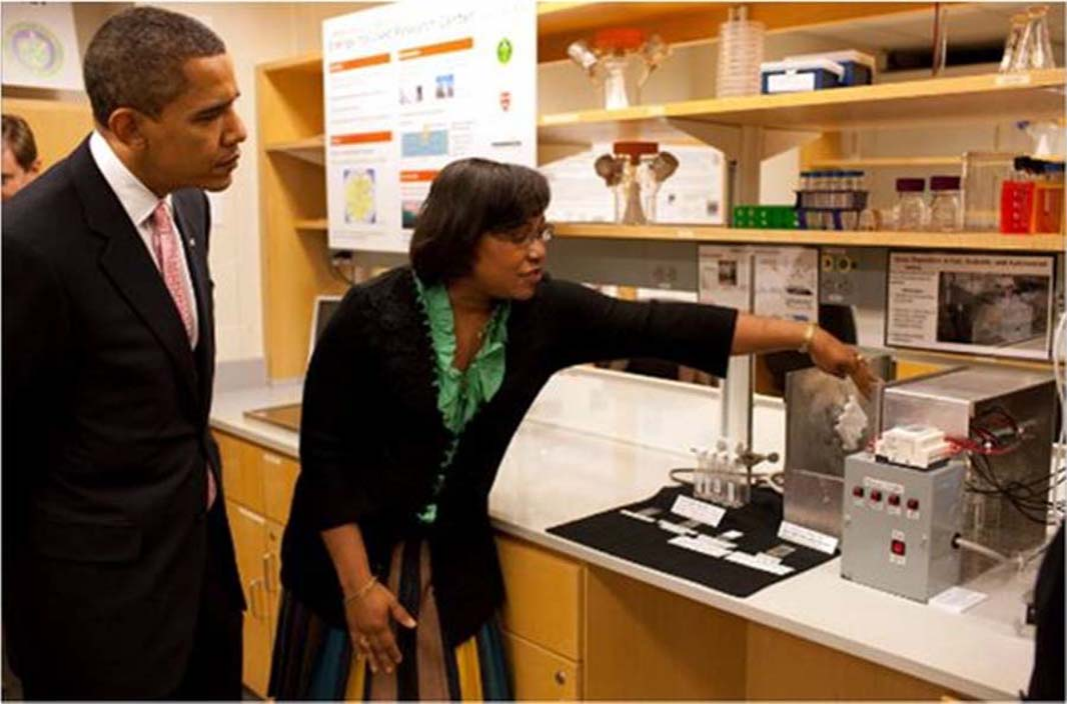
Professor Hammond describing the spray layer‐by‐layer technology to President Barack Obama during the President's visit to MIT on October 23, 2009 *Source.* MIT Spring 2010 Chemical Engineering Newsletter (http://web.mit.edu/cheme/alumni/newsletter/XCurrentsSpring10.pdf)

**Figure 2:**
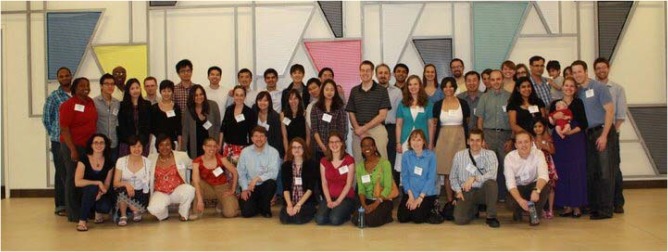
Group photo at the 15 year Hammond Lab reunion on June 4, 2011 Photo courtesy of the Hammond Group

Professor Hammond's hallmark is her infectious enthusiasm and optimism, which are always inspiring and create a lasting impression. She is a champion of her students, always eager to engage and provide guidance, discuss research, and embrace new ideas. Professor Hammond has fostered an intellectually stimulating, open, positive, and collegial research environment in the laboratory and has been a role‐model mentor, devoted teacher, and colleague to her many trainees. On their behalf, I express my admiration for her scientific contributions and appreciation for the opportunity to learn from her experience. We look forward to embracing and applying her values and pedagogy to our careers.

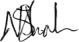
